# Stimulating GABAergic Neurons in the Nucleus Accumbens Core Alters the Trigeminal Neuropathic Pain Responses in a Rat Model of Infraorbital Nerve Injury

**DOI:** 10.3390/ijms22168421

**Published:** 2021-08-05

**Authors:** Jaisan Islam, Elina KC, Soochong Kim, Hyong Kyu Kim, Young Seok Park

**Affiliations:** 1 Department of Medical Neuroscience, College of Medicine, Chungbuk National University, Cheongju 28644, Korea; jaisanislam92@gmail.com (J.I.); elnachhetri13.ec@gmail.com (E.K.); 2 ISCRM, Department of Veterinary Medicine, College of Veterinary Medicine, Chungbuk National University, Cheongju 28644, Korea; skim0026@chungbuk.ac.kr; 3 Department of Medicine and Microbiology, College of Medicine, Chungbuk National University, Cheongju 28644, Korea; hkkim69@chungbuk.ac.kr; 4 Department of Neurosurgery, Chungbuk National University Hospital, Cheongju 28644, Korea

**Keywords:** microdialysis, nucleus accumbens core, optogenetics, trigeminal neuralgia, VPM thalamus

## Abstract

The nucleus accumbens core (NAcc) is an important component of brain reward circuitry, but studies have revealed its involvement in pain circuitry also. However, its effect on trigeminal neuralgia (TN) and the mechanism underlying it are yet to be fully understood. Therefore, this study aimed to examine the outcomes of optogenetic stimulation of NAcc GABAergic neurons in an animal model of TN. Animals were allocated into TN, sham, and control groups. TN was generated by infraorbital nerve constriction and the optogenetic virus was injected into the NAcc. In vivo extracellular recordings were acquired from the ventral posteromedial nucleus of the thalamus. Alterations of behavioral responses during stimulation “ON” and “OFF” conditions were evaluated. In vivo microdialysis was performed in the NAcc of TN and sham animals. During optogenetic stimulation, electrophysiological recordings revealed a reduction of both tonic and burst firing activity in TN animals, and significantly improved behavioral responses were observed as well. Microdialysis coupled with liquid chromatography/tandem mass spectrometry analysis revealed significant alterations in extracellular concentration levels of GABA, glutamate, acetylcholine, dopamine, and citrulline in NAcc upon optic stimulation. In fine, our results suggested that NAcc stimulation could modulate the transmission of trigeminal pain signals in the TN animal model.

## 1. Introduction

Trigeminal neuralgia (TN) or “*tic douloureux*” can present as an extreme form of chronic neuropathic pain and include a spontaneous and exaggerated painful response alongside a negative affective, motivational, and cognitive state [1,2]. Chronic constriction injury of the infraorbital nerve (CCI-ION) is an established model for TN experiments, as this model exhibits the principal TN syndromes. Although the typical form of TN responds satisfactorily to surgical interventions, the atypical form of TN does not [2,3]. To overcome this and find an efficacious solution, maladaptive neural circuits linked with different states in the brain have been widely studied.

The limbic circuit of the basal ganglia, centered in the nucleus accumbens (NAc), is known as the pleasure center of the brain and regulates reward- and aversion-based behavior. However, an important aspect of the NAc, where approximately 95% of the neurons are GABAergic medium spiny neurons (MSNs) [4], is often overlooked, which is its significant intervention in pain regulation by modulating its projections of medium spiny neurons to get positive feelings and effects of reward from pain relief [5]. Alteration in the gray matter of the NAc was observed in TN. Through its projection to the PAG-RVM and brainstem, NAc may affect behavioral outcomes by manipulating the emotional aspects of pain through the descent of the dopaminergic pathway and GABAergic input to the other brain areas [6,7,8,9,10]. Between the two regions of the NAc, the NAc core (NAcc) has been identified to have more involvement and a dominant role in nociception over the NAc shell (NAcsh) [11,12]. The NAcc integrates the mesencephalic dopaminergic signals from the VTA and SN, as well as glutamatergic information from the PFC, amygdala, thalamus, prelimbic cortex, hippocampus, and subiculum [5,9]. Therefore, to analyze the effects of stimulating NAcc in a TN condition, we used an optogenetic stimulation approach with the human synapsin promoter to increase the activity of GABAergic neurons in NAcc, as it is well-established that pptogenetics is suitable for understanding the functional dissection of neural circuits that manipulate the neural substrates of behavior, as it can achieve more precise temporal and spatial control over specific neural populations than pharmacological or electrical interventions [7,13,14].

Present approaches available for atypical trigeminal neuropathic pain management have not been able to deliver fruitful long-term therapeutic efficacy. Besides, several medications have revealed unexpected side effects, such as overdose and addiction. These setbacks necessitate a search for alternative potential options for orofacial pain management. In addition, studies have revealed the modulatory effect of NAcc in attenuating chronic spinal back pain. Therefore, the purpose of this study was to see if changing the activity of GABAergic NAcc neurons provides analgesic feedback in the event of orofacial pain in a CCI-ION rat model.

## 2. Results

### 2.1. Changes in Pain Behaviors Following ION Ligation

**Figure 1:**
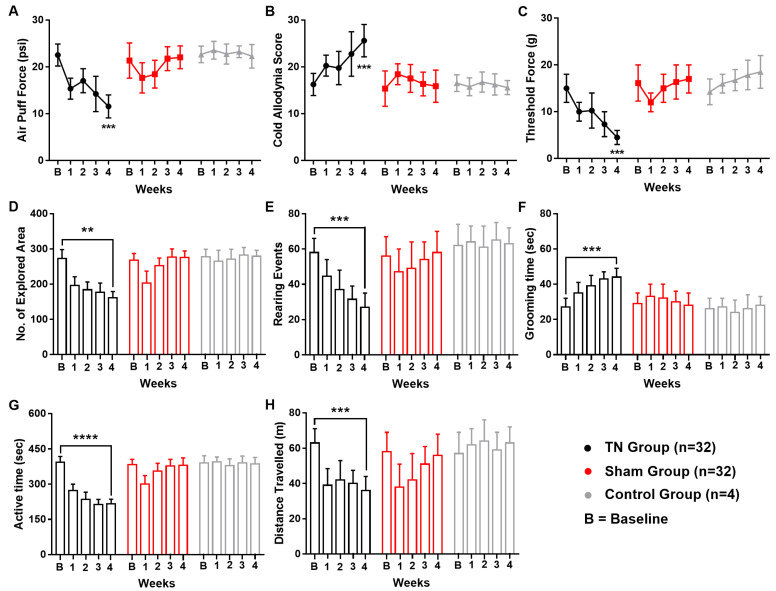
Changes in pain behaviors following ION ligation. Behavioral scores of the TN, sham, and control groups (**A**–**H**). (**A**) Results from the air-puff test for the ipsilateral trigeminal facial area. (**B**) Cold allodynia score results for the ipsilateral facial area with acetone drops. (**C**) Mechanical allodynia (von Frey test) results for the ipsilateral facial side. (**D**–**H**) Results of the open field test: (**D**) number of explored areas, (**E**) rearing events, (**F**) grooming time, (**G**) active time, and (**H**) distance traveled. **, *p* < 0.01; ***, *p* < 0.001; ****, *p* < 0.0001, significant difference determined using an analysis of variance. Data are represented as means ± SD.

Following ION ligation, the behavioral test scores of the air-puff test, cold allodynia score test, and mechanical allodynia test suggested the presence of chronic pain in the TN animals. In contrast, no significant changes were observed in the behavioral scores of the sham and control animals. In the TN group, a two-way ANOVA revealed significant, gradual decreases in the air-puff test scores over the four weeks (22.544 ± 2.354 psi to 11.560 ± 2.450 psi; two-way ANOVA, *F* (2, 325) = 68.61, *p* < 0.001; Figure 1A). In addition, the facial cold allodynia scores significantly increased from 16.25 ± 2.354 to 25.620 ± 3.450 (two-way ANOVA, *F* (2, 325) = 32.26, *p* < 0.001; Figure 1B). The mechanical allodynia scores also significantly decreased from 15 g to 4.5 ± 1.5 g following surgery (two-way ANOVA, *F* (2, 325) = 82.78, *p* < 0.001; Figure 1C).

In the open field test, significant alterations in the exploration rate (*p* < 0.01, Figure 1D), rearing events (*p* < 0.001, Figure 1E), grooming time (*p* < 0.001, Figure 1F), active time (*p* < 0.0001, Figure 1G), and overall distance travelled (*p* < 0.001, Figure 1H) were observed in TN animals but not in the sham and control group. All these observations from the open field test result analysis pointed towards a sequential relationship between orofacial pain and social anxiety in our experimental model animals.

### 2.2. Extracellular Recording Data In Vivo

**Figure 2:**
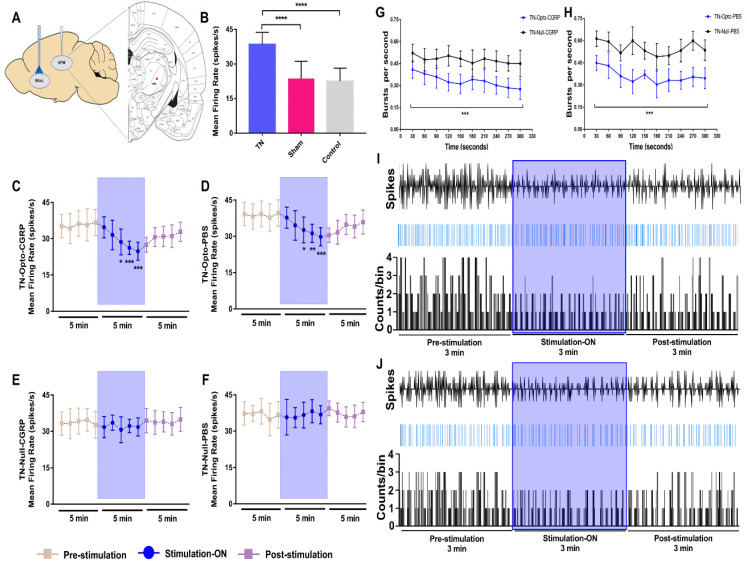
Extracellular recording data in vivo. (**A**) Schematic diagram showing extracellular recording area. (**B**) Evoked firing rates in VPM neurons in TN (*n* = 32) animals versus sham (*n* = 32) and control (*n* = 4) animals (****, *p* < 0.0001, significant difference determined using unpaired *t*-tests). (**C**,**D**) In vivo recordings of the VPM thalamus from TN-Opto animals. (**C**) Findings for the TN-Opto-CGRP (*n* = 8) group. (**D**) Findings for the TN-Opto-PBS (*n* = 8) group. A decrease in firing output (spikes/s) was observed during the stimulation “ON” period. (**E**,**F**) No changes in the VPM thalamus firing rates were observed in the TN-Null-CGRP (*n* = 8) or TN-Null-PBS (*n* = 8) groups (two-way analysis of variance *, *p* < 0.05; **, *p* < 0.01; ***, *p* < 0.001). (**G**,**H**) Changes in the burst firing rates of the TN animals following optogenetic stimulation. A significant reduction was observed in the TN-Opto-CGRP (**G**, blue line) and TN-Opto-PBS (**H**, blue line) groups. ***, *p* < 0.001, analysis of variance. (**I**,**J**) Peri-event raster histogram of VPM neuron responses show spike traces (above), raster traces (middle), and rate histogram (below) in the TN-Opto-CGRP (**I**) and TN-Opto-PBS (**J**) groups in optic stimulation “ON and “OFF” states. Bin size = 50 ms. VPM thalamic responses were decreased during optic stimulation (blue area).

We examined the firing rates of VPM neurons (Figure 2A) in different conditions in our study. In the TN, sham, and control groups, after model-making surgery, significantly higher firing rates were observed in the TN-lesioned (*n* = 32) rats (38.095 ± 4.233 spikes/s) compared to sham (*n* = 32) (23.503 ± 7.314 spikes/s, unpaired *t*-test (*t*, df) = (9.171, 62), *p* < 0.001) and control (*n* = 4) rats (22.564 ± 5.492 spikes/s, unpaired *t*-test (*t*, df) = 5.686, 34), *p* < 0.001) (Figure 2B).

We assessed the effects of optical stimulation on NAcc in the TN-Opto and TN-Null groups using in vivo extracellular recordings both with and without α-CGRP receptor pathway blockade conditions. We used the α-CGRP receptor antagonist in a positive control manner during extracellular recording because it has already been proved that the activity of α-CGRP neurons is necessary for neuropathic hypersensitivity spontaneous pain and it can excite second-order neurons in the central sensitization of the trigeminal nucleus caudalis (TNC) [15,16].

Optogenetic stimulation induced significant changes in VPM neuron activity in the TN-Opto group. The spike rates in the pre-stimulation, stimulation “ON”, and post-stimulation conditions in the TN-Opto-CGRP (*n* = 8) group were 35.6276 ± 5.70, 29.203 ± 4.472, and 30.5914 ± 3.961, respectively. A two-way ANOVA revealed significant effects of optic stimulation (*F* (2165) = 29.25, *p* < 0.001) (Figure 2C). The spike rates for the pre-stimulation, stimulation “ON”, and post-stimulation conditions in the TN-Opto-PBS (*n* = 8) group were 38.8276 ± 5.3074, 33.203 ± 4.672, and 34.1914 ± 4.361, respectively (*F* (2165) = 23.04, *p* < 0.001) (Figure 2D). No significant changes in the thalamic firing rates were observed in the TN-Null-CGRP (*n* = 8) or TN-Null-PBS (*n* = 8) groups (Figure 2E,F).

The burst rates also appeared to decrease during optogenetic stimulation in the TN-Opto group. The thalamic burst rates for the pre-stimulation, stimulation “ON”, and post-stimulation conditions in the TN-Opto-CGRP group were 0.44 ± 0.08/s, 0.28 ± 0.06/s, and 0.40 ± 0.10/s, respectively. A two-way ANOVA revealed significant effects of optogenetic stimulation (*F* (2276) = 5.45, *p* < 0.05) (Figure 2G). Optogenetic stimulation also significantly decreased the burst rates in the TN-Opto-PBS group. The thalamic burst rates for the pre-stimulation, stimulation “ON”, and post-stimulation conditions in the TN-Opto-PBS group were 0.75 ± 0.07/s, 0.58 ± 0.06/s, and 0.67 ± 0.10/s, respectively. A two-way ANOVA revealed significant effects of optogenetic stimulation (*F* (2276) = 3.57, *p* < 0.05) (Figure 2H). We did not observe any alterations in the burst rate of the TN-Null animals. In both the TN-Opto-CGRP and TN-Opto-PBS groups, we observed greater thalamic response inhibition during the stimulation “ON” state than during the stimulation “OFF” state (Figure 2I,J).

These results suggest that optogenetic stimulation of the NAcc produced analgesia and, as a result, significant improvement in VPM thalamic activity both with and without α-CGRP receptor blockade conditions. No effects of laser stimulation on the thalamic firing patterns were observed in the TN-Null-CGRP and TN-Null-PBS groups.

### 2.3. Attenuation of Hyperalgesia by Optogenetic Stimulation

**Figure 3:**
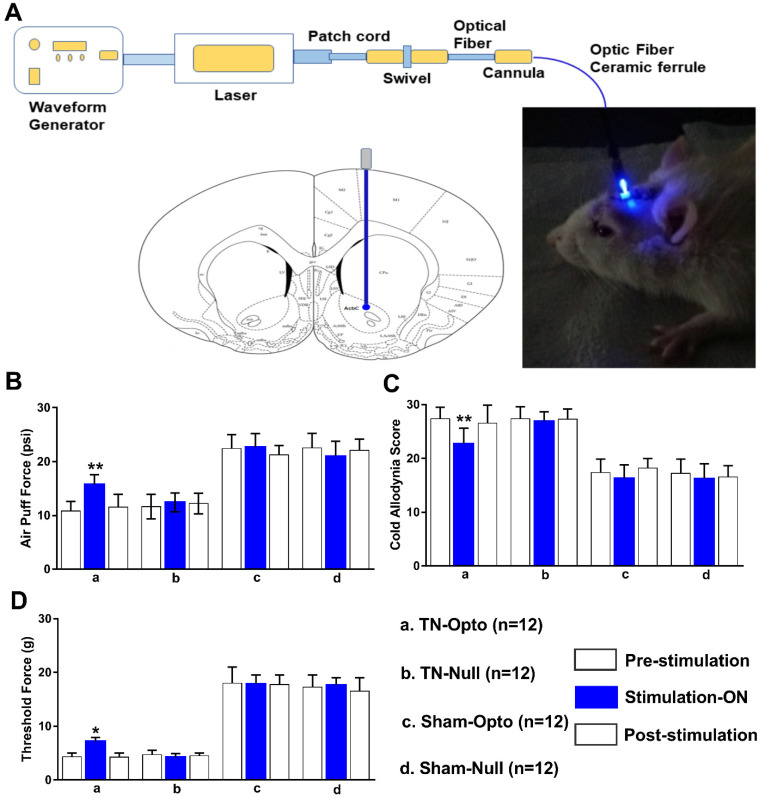
Attenuation of hyperalgesia by optogenetic stimulation. (**A**) Optogenetic stimulation setup and area. (**B**–**D**) Changes in behavioral responses following blue laser stimulation: results from the air-puff test (**B**), cold allodynia score test (**C**), and von Frey test (**D**) of TN-Opto (*n* = 12) (a), TN-Null (*n* = 12) (b), Sham-Opto (*n* = 12) (c), and Sham-Null (*n* = 12) (d) animal groups, respectively. Only the TN-Opto group exhibited significant changes in behavioral scores during the stimulation “ON” condition. No significant changes in behavioral scores were observed in other animal groups during the stimulation “ON” condition. *, *p* < 0.05; **, *p* < 0.01, significant differences determined using an analysis of variance. Data are represented as means ± SD.

Optogenetic stimulation approach of NAcc (Figure 3A) induced significant changes in the behavioral scores in the TN group. TN-Opto (*n* = 12) animals exhibited significant alterations in behavioral responses in the air-puff test during the stimulation “ON” condition. For these animals, a one-way ANOVA revealed a significant effect of optogenetic modulation (pre-stimulation: 10.84 ± 1.75 psi; stimulation “ON”: 15.73 ± 1.85 psi; post-stimulation: 11.56 ± 2.356 psi; *F* (2,33) = 29.35, *p* < 0.01) (Figure 3B(a)). Significant decreases in the cold allodynia score were also observed during optogenetic stimulation (pre-stimulation: 27.35 ± 2.15; stimulation “ON”: 22.73 ± 2.85; post-stimulation: 26.56 ± 3.356; one-way ANOVA, *F* (2,33) = 24.59, *p* < 0.01) (Figure 3C(a)). The mechanical allodynia scores also improved in TN-Opto animals when stimulated optogenetically (pre-stimulation: 4.35 ± 0.65 g; stimulation “ON”: 7.15 ± 0.75 g; post-stimulation: 4.25 ± 0.70 g; one-way ANOVA, *F* (2,33) = 15.454, *p* < 0.05) (Figure 3D(a)).

The other animal groups (TN-Null, Sham-Opto, Sham-Null) exhibited no significant changes in behavior under optogenetic stimulation conditions (Figure 3B(b–d),C(b–d),D(b–d)).

**Figure 4:**
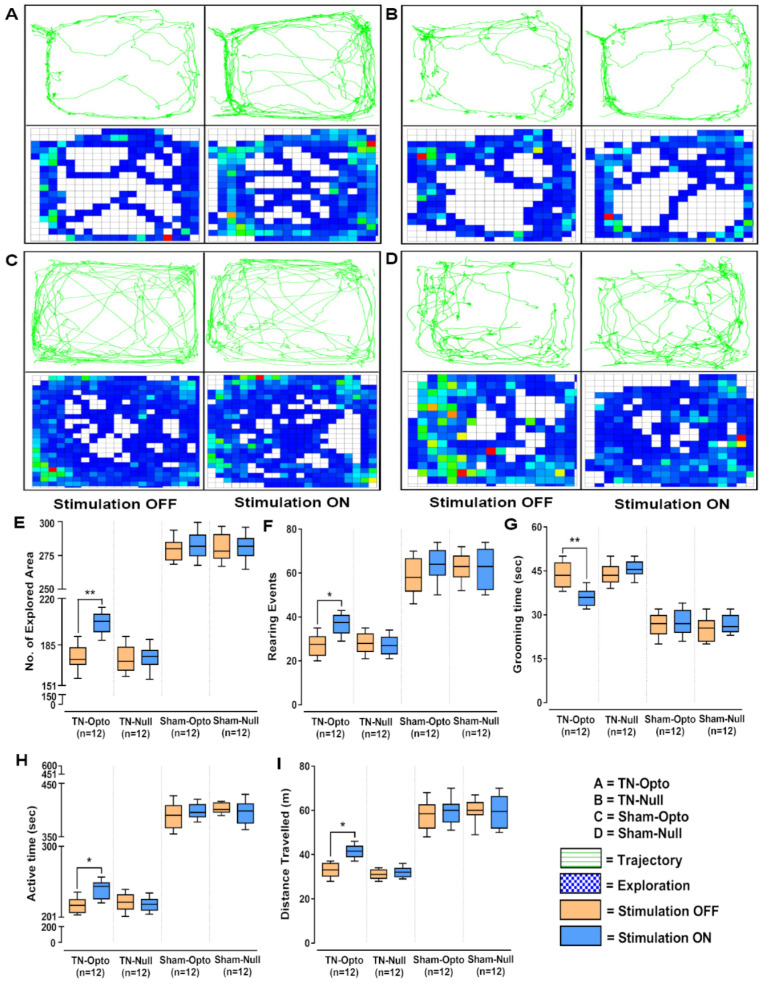
Alteration of behavioral responses in the open field test by optogenetic stimulation. (**A**–**D**) Trajectory and exploration representations of the TN-Opto, TN-Null, Sham-Opto, and Sham-Null groups, respectively, during stimulation “OFF” and “ON” conditions. (**E**) Numbers of explored areas, (**F**) rearing events, (**G**) grooming time, (**H**) active time, and (**I**) distance traveled. In all the behavioral paradigms, the TN-Opto group exhibited significant alterations with the stimulation “ON” condition but no significant changes in behavioral scores were observed in other animal groups with the stimulation “ON” condition. *, *p* < 0.05; **, *p* < 0.01, significant differences determined using unpaired *t*-test.

In the open field test also, NAcc optic modulation provided significantly improved behavioral responses in all the analyzed parameters in the TN-Opto group but not in the TN-Null, Sham-Opto, and Sham-Null groups. In TN-Opto group animals, optogenetic stimulation provoked changes in trajectory and exploratory manners (Figure 4A). In contrast, the other three groups did not exhibit any significant variation in their trajectory or exploratory manners during optic stimulation compared to the stimulation “OFF” condition (Figure 4B–D). TN-Opto grouped animals showed increased numbers of explored areas (unpaired *t*-test, *p* < 0.01; Figure 4E) and rearing events (unpaired *t*-test, *p* < 0.05; Figure 4F) and decreased grooming time (unpaired *t*-test, *p* < 0.01; Figure 4G) during optogenetic stimulation. They also exhibited improvements in activity time (unpaired *t*-test, *p* < 0.05; Figure 4H) and overall distance traveled (unpaired *t*-test, *p* < 0.05; Figure 4I) with optic stimulation compared to the stimulation “OFF” condition.

These findings support our hypothesis that stimulation of the NAcc exerted an antinociceptive effect on the TN condition and hence generated positive behavioral response alterations in TN-Opto grouped animals.

### 2.4. Effect of Optogenetic Stimulation on Extracellular Concentration Level of GABA, Glutamate, Dopamine, Acetylcholine, and Citrulline in NAcc

**Figure 5:**
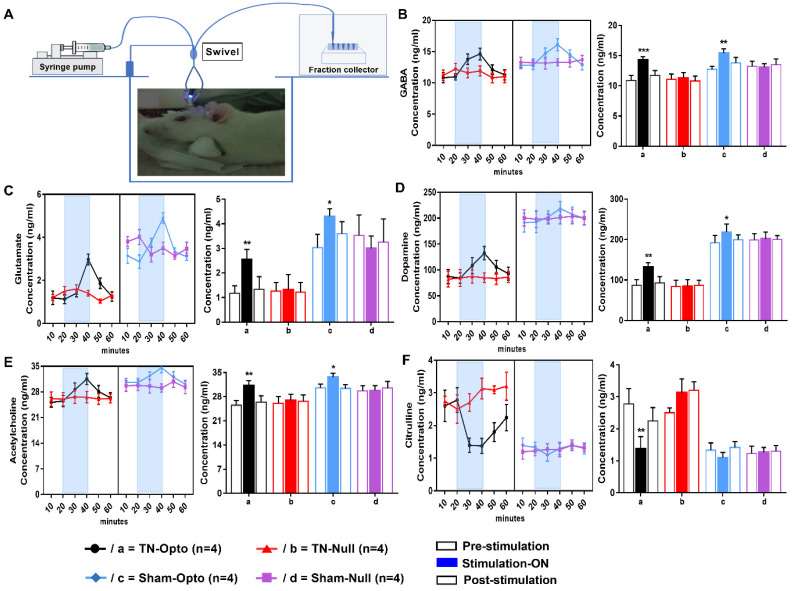
Effect of optogenetic stimulation on the extracellular concentration level of GABA, glutamate, dopamine, acetylcholine, and citrulline in NAcc. (**A**) Microdialysis setup and procedure of microdialysate collection. Extracellular conc. level (ng/mL) of GABA (**B**), glutamate (**C**), dopamine (**D**), acetylcholine (**E**), and citrulline (**F**) throughout the 60 min duration (pre-stimulation: 0–20 min; stimulation “ON”: 20–40 min; post-stimulation: 40–60 min). In both TN-Opto (*n* = 4) and Sham-Opto (*n* = 4) groups, alterations in the conc. levels of all the five neurotransmitters were observed during optogenetic stimulation but not in the TN-Null (*n* = 4) and Sham-Null (*n* = 4) animal groups. *, *p* < 0.05; **, *p* < 0.01; ***; *p* < 0.001, significant differences determined using an analysis of variance. Data are represented as means ± SD.

After performing in vivo microdialysis (Figure 5A), we found that optic stimulation caused significant change in the concentration (conc.) level (ng/mL) of our selected neurotransmitters compared to pre- and post-stimulation conditions in the TN-Opto and Sham-Opto groups. In TN-Opto (*n* = 4) grouped animals, increased GABA levels (pre-stimulation: 10.90 ± 0.81 ng/mL; stimulation “ON”: 14.325 ± 0.51 ng/mL; post-stimulation: 11.70 ± 0.82 ng/mL; one-way ANOVA, *F* (2,9) = 37.54, *p* < 0.001, Figure 5B(a)), increased glutamate levels (pre-stimulation: 1.17 ± 0.31 ng/mL; stimulation “ON”: 2.55 ± 0.41 ng/mL; post-stimulation: 1.33 ± 0.52 ng/mL; one-way ANOVA, *F* (2,9) = 24.79, *p* < 0.01, Figure 5C(a)), increased dopamine levels (pre-stimulation: 87.14 ± 13.54 ng/mL; stimulation “ON”: 132.27 ± 10.04 ng/mL; post-stimulation: 92.82 ± 15.66 ng/mL; one-way ANOVA, *F* (2,9) = 21.792, *p* < 0.01, Figure 5D(a)), increased acetylcholine levels (pre-stimulation: 25.52 ± 1.34 ng/mL; stimulation “ON”: 31.18 ± 1.51 ng/mL; post-stimulation: 26.44 ± 1.84 ng/mL; one-way ANOVA, *F* (2,9) = 19.738, *p* < 0.01, Figure 5E(a)), and decreased citrulline levels (pre-stimulation: 2.78 ± 0.48 ng/mL; stimulation “ON”: 1.38 ± 0.38 ng/mL; post-stimulation: 2.24 ± 0.42 ng/mL; one-way ANOVA, *F* (2,9) = 18.57, *p* < 0.01, Figure 5F(a)) were found during the blue laser “ON” condition. Sham-Opto (*n* = 4) grouped animals also showed significant alterations in extracellular conc. levels of all neurotransmitters except for citrulline. In animals of the TN-Null (*n* = 4) and Sham-Null (*n* = 4) groups, the extracellular conc. levels of these neurotransmitters did not show any significant changes in the stimulation “ON” condition.

These data disclose that stimulation of NAcc caused alteration in the release of neurotransmitters among the reward circuitry, which ultimately resulted in the altered conc. levels of neurotransmitters in the TN-Opto and Sham-Opto groups.

### 2.5. Viral Expression, Immunostaining of Cannula Placement, and Neuronal Activation in the NAcc

**Figure 6:**
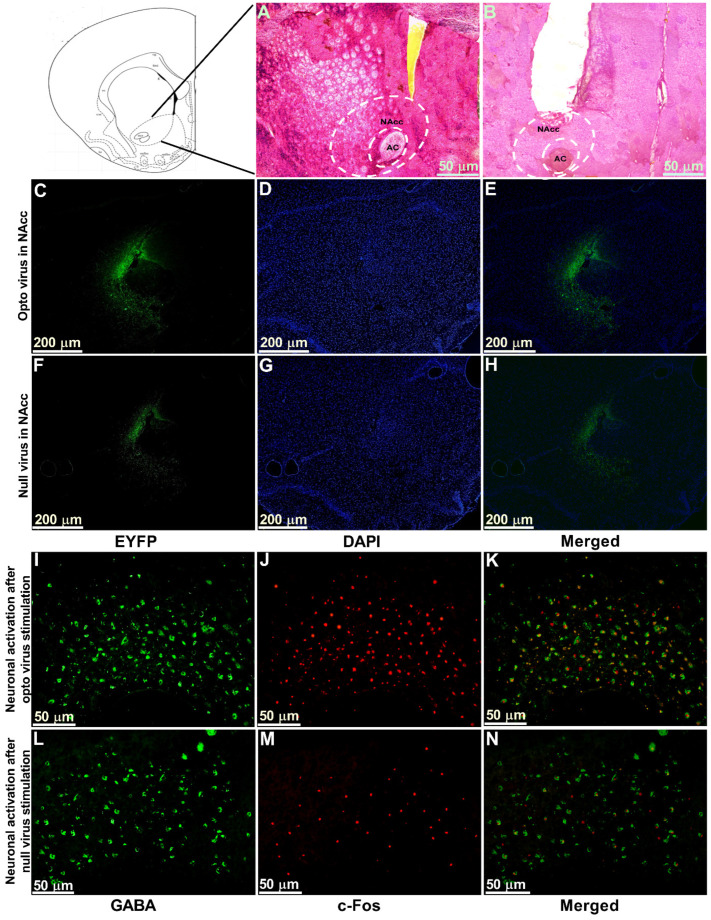
Viral expression, immunostaining of cannula placement, and neuronal activation in the NAcc. (**A**) H&E staining of brain tissue section showing the anatomical location of NAcc. (**B**) H&E staining of brain tissue section showing the location of optic fiber and guide cannula placement in NAcc. Scale bar = 50 µm. (**C**–**E**) Optogenetic viral expression in the NAcc of TN-Opto animals. (**F**–**H**) Null viral expression in the NAcc of TN-Null animals. (**C**,**F**) EYFP, (**D**,**G**) DAPI, and (**E**,**H**) merged. Scale bar = 200 µm. (**I**–**K**) Colocalization of staining for c-Fos and GABA by double immunofluorescence labeling in optogenetic virus-injected animal. (**L**–**N**) Colocalization of staining for c-Fos and GABA by double immunofluorescence labeling in null virus-injected animal. Double immunoreactivity for GABA (**I**,**L**), c-Fos (**J**,**M**), and merged c-Fos and GABA (**K**,**N**) in NAcc. Scale bar = 50 µm.

The optogenetic target for the present study was the NAcc, which was stereotaxically located using a rat atlas. Hematoxylin and eosin (H&E) staining was performed to locate the anatomical position of NAcc (Figure 6A) and the cannula placement in the animals (Figure 6B). We observed the expression of optogenetic virus (Figure 6C–E) and null viruses (Figure 6F–H) in the contralateral NAcc using immunofluorescence. Viral expression in neurons stained with EYFP and DAPI was obtained by using a fluorescence microscope and ImageJ software. Further, to confirm that stimulated optogenetic virus caused activation of GABAergic neurons in the NAcc, we performed double immunofluorescence labeling for GABAergic neurons (Figure 6I) and c-Fos positive cells (Figure 6J). We observed whether Fos expression ensued in GABAergic neurons and found the colocalization of the c-Fos immunoreactivity and GABAergic neurons (Figure 6K), which indicated the activation of GABAergic neurons in optogenetic virus-injected animals. In contrast, there was very little c-Fos expression found in the NAcc of the null virus-injected animals (Figure 6L–N).

## 3. Discussion

In our study, we observed that optogenetic stimulation of the contralateral NAcc altered the TN pain behavioral response, the extracellular concentration level of neurotransmitters in the NAcc, and the VPM thalamic output in a rat model of CCI-IoN. Our findings provide strong evidence to support the hypothesis that modulation of NAcc activity could be an intriguing part of TN pain management.

TN is an severe episodic pain condition of the orofacial region associated with injury or dysfunction of the fifth cranial nerve or its ganglion and also associated with depression. The excitability threshold of affected fibers can be decreased by several conditions, resulting in improper sensory propagation toward the surrounding fibers. These sensory nerve fibers are mainly Aδ- and C-fibers, and their cell bodies are rooted in the trigeminal ganglion (TG). Nociceptive signals are conveyed through these trigeminal afferents and projected to the trigeminal spinal caudalis (Vc) nucleus of the brainstem, where they synapse with the second-order neurons and are projected to the somatosensory and limbic cortices via reciprocal aberrant sensory signals assembled in the sensory area of the thalamus (i.e., the VPM), thereby initiating pain sensation and behavioral changes [2,17,18,19,20,21]. Similar findings were observed in our study, with TN animals exhibiting painful behavioral responses in all behavior tests. In contrast, the sham and control groups were consistent throughout the study period.

Optical neuromodulation offers several promising technological advantages. The optical neural interface raises the prospect of specific interventions in a specific manner with millisecond temporal precision. This light-assisted approach to neuron stimulation eliminates the necessity of placing electrodes within a relatively homogenous group of brain nuclei, which is challenging [13,22,23,24]. Our optogenetic modulation relied on the heterologous expression of the photosensitive actuator channelrhodopsin-2 (ChR2), which is activated by blue light (~473 nm). The linkage between optical excitation and activation of neurons mediated by ChR2 was used as a potent tool for increasing cytoplasmic Ca^2+^ concentrations [25,26]. NAcc is mostly (95%) composed of GABAergic MSNs containing dopaminergic receptors. There are also local circuit interneurons (cholinergic and GABAergic) present in the NAcc [27]. When blue light was introduced into NAcc where AAV-hSyn-ChR2-EYFP had been transfected, it caused excitation of the GABAergic neurons, which was observed in our immunofluorescence results. As glutamatergic input from PFC to NAcc is decreased and presynaptic GABAergic neurotransmitter release at the synapses of NAc MSNs is increased in chronic neuropathic pain, researchers have shown that positive modulation of GABA and speeding up of the regular activity of reward circuitry by optogenetic stimulation resulted in pain modulation effects in neuropathic pain studies [7,21,28,29,30,31]. Furthermore, positive activation of the NAcc in response to the offset of noxious stimuli has been observed in functional MRI [32]. It has also been shown to have antidepressant properties, which ultimately result in reward perception [6,33,34]. However, in contrast to these studies, Chang et al. (2014) reported pain reduction in a spared nerve injury rat model after inhibiting the activity of NAcsh by lidocaine [35]. The discrepancy with this finding could be linked to the variations due to plastic changes in the neural circuitry of NAcc and NAcsh.

Along with GABAergic neurons, the interneurons, which act as feedforward substrates, were also activated. Cholinergic interneurons are the sources of acetylcholine (Ach) production [27] and, in our study, we found an elevated concentration level of Ach during optogenetic stimulation. GABAergic interneurons, which comprise less than 1% of the accumbens neurons, play an important role in the presynaptic and postsynaptic transmissions [27] by mediating local inhibition. The input–output ratio of NAc becomes disproportional in conditions of pain [30]. Therefore, increased activity of these interneurons helps in the restoration of the intrinsic circuitry. However, MSNs have a regulatory effect on the activity of this local circuitry and reduced activity of MSNs can be noted in pain conditions, which causes alterations in the action of interneurons. Therefore, the increased activity of MSNs enables them to modulate the interneurons and maintain the optimum intrinsic circuitry of the NAcc.

Analysis of records from recent studies on preclinical animal models and human patients shows that mesolimbic reward circuitry reorganization has long been hypothesized to be involved in pain chronification and pain-related emotional aspects [29,36,37]. The NAcc, an important part of reward circuitry, forms reciprocal projections with regions critical for pain and depression, such as the PFC, hippocampus, amygdala, and VTA. The NAcc also has dense projections to the ventral pallidum, globus pallidus, and substantial nigra, which have significant importance for the manifestation of motivated behavior. These areas can modulate the thalamus through direct and indirect projections to the thalamus [5,9,38]. Earlier studies revealed that the NAcc has more prominent involvement with chronic neuropathic pain pathology [5,7,10,11,12,39]. Midbrain dopaminergic neurons increase their activity in response to reward cues and reduce their activity on aversive stimuli [38,39,40,41,42]. Functional neuroimaging has shown correlated dynamism between the NAcc and other regions engaged in sensory and emotional aspects of pain perception and its modulation [39,43,44]. NAcc is one of the few locations where GABAergic dopamine receptors and glutamate neurotransmission converge. This unrivaled unique neuroanatomic position enables the NAcc to modulate pain processing directly through the connections mentioned above. These connections allow the NAcc to form inhibitory projections to the medial thalamus, which in turn project to the motor cortex and some of the ACC and dorsal horn neurons [5,45,46]. Through this striatothalamocortical circuitry and bidirectional loop of pain modulation among the PAG, the habenula, and nucleus accumbens by opioid regulation, NAcc stimulation can produce analgesia by closing the pain sensory gate. It can modulate the pre-cortical and subcortical regions’ neural activity and enable the NAcc to influence behavioral outcomes and the affective (unpleasant) component of pain in the presence of reward cues [5,6,7,47,48,49]. Several animal studies have discussed the role of the inputs and outputs of the NAcc in nociception. After stimulating the NAcc, our results also support existing reports on the involvement of the NAcc in neuropathic pain [6,7,29,50,51]. Animals showed improved behavioral responses in behavior tests under the blue laser “ON” condition.

We performed in vivo extracellular recording from the contralateral VPM thalamus. We observed the onset of hyperactivity in the VPM thalamic response after CCI-IoN surgery compared to sham and control animals. TN causes altered thalamocortical rhythms that result in increased sensitivity to pain and pain generation in response to non-noxious stimuli [52]. The spinal trigeminal nucleus conveys craniofacial somatosensory information to the thalamus from the brainstem. Earlier studies also showed that TN is associated with anatomical and biochemical changes in the VPM thalamus that cause thalamic tonic firing hyperresponsiveness and an increase in the burst firing rate [1,2,53,54,55]. Since stimulation of the NAcc causes alteration in its robust interconnections with areas involved in pain circuitry, manipulation of these networks produces antinociception with downstream effects. Therefore, during optogenetic stimulation of NAcc in our study, a significant reduction of VPM thalamic hyperresponsiveness was observed as a consequence of alleviated pain sensation [29,56,57].

Earlier studies involving in vivo microdialysis have disclosed that extracellular concentration levels of various neurotransmitters change upon the pain condition in the NAc. Therefore, alteration of the balance of relevant neurotransmitters in different brain regions happens in chronic pain conditions and, because these alterations lead to an incited central pain processing pathway, we measured the deflection in the extracellular concentration levels of glutamate, dopamine, acetylcholine, and citrulline in optic stimulation “off” and “on” conditions when the release of GABA is increased due to the excitation of GABAergic neurons. In chronic pain conditions, extracellular concentration levels of dopamine, glutamate, and acetylcholine have been found to be decreased in NAcc compared to normal conditions and, in alleviated pain sensation, which acts as a reward-getting semiotic, their concentration levels are enhanced, which has modulatory effects on the neural reward circuitry as well [5,6,10,29,58,59,60]. The extracellular level of citrulline appeared to be increased upon nociceptive stimuli, whereas abatement of its manifestations was observed after pain-relieving and reward-getting cues [61,62]. Excitation of GABAergic neurons and cholinergic interneurons resulted in increased GABA and ACh levels and these changes affected the alterations of other neurotransmitters [63]. NAc gets dopamine and glutamate from extrinsic sources, which are decreased during pain. Since activation of NAcc caused interference in the pain processing pathway by modulating reward circuitry, the inputs from glutamate projections and dopamine projections into the Nacc are increased in response to an alleviated pain condition. In our microdialysis analysis, we also observed similar positive feedback alterations for glutamate, dopamine, acetylcholine, and citrulline during optic stimulation conditions in both the TN-Opto and Sham-Opto groups

The current study had several limitations. Postoperative stress might influence pain chronicity, although all the animals were taken care of well after surgery. We did not perform extracellular recording from the NAcc itself, and anesthesia could have affected the thalamic firing rates.

## 4. Materials and Methods

### 4.1. Ethics Approval

All experiments were conducted under the guidelines of the Animal Care and Use Committee (IACUC) of Chungbuk National University (CBNUA-1346-20-02) in Korea. All surgical procedures were performed under general anesthesia (with an intraperitoneal injection of 15 mg/kg of Zoletil (Zoletil50^®^, Virbac Laboratories, Carros, France) and 9 mg/kg of Rompun (Rompun^®^, Bayer, Seoul, South Korea) in saline) and animals were observed daily after surgery. Animals were carefully handled before and during the experiments to lessen their affliction. All animal experiments were performed during the light period and at the same time in the Laboratory Animal Research Center of Chungbuk National University.

### 4.2. Experimental Subjects

The present study included a total of 68 adult female Sprague–Dawley rats (age: 8 weeks; Koatech, Pyeongtaek, Korea), each weighing 200–250 g on arrival. All the animals were maintained at constant temperature (22 ± 1 °C) and humidity (50–60%) with *ad libitum* access to fresh food and water under a 12:12 h light and dark cycle. In our study, we chose to use female animals because it has been well-established that TN-associated disorders are more prevalent among females than males and that CGRP expression is found to be much higher in females than in males [15]. All tests were conducted in a randomized, double-blind, controlled manner. The animals were randomly grouped into a TN group (*n* = 32), sham group (*n* = 32), and control group (*n* = 4). The timeline of the experimental protocol is shown in Figure S1A.

### 4.3. CCI-ION Model-Making and Optogenetic or Null Virus Injection

To generate the TN model, CCI-ION surgery was performed in 32 animals as previously described [2,64] (Figure S2). Briefly, after achieving general anesthesia, the animals were mounted onto the surgical field in a prone position. An incision was made along the curve of the frontal bone above the left eye to reveal the ION. After revealing the ION, two ligatures were gently placed 3–4 mm apart. Finally, the incision above the eye was sutured with silk (3–0). Sham animals (*n* = 32) underwent the same procedures, except for the ligature placement. After surgery, the rats were given fresh pelleted food and water *ad libitum* and checked for survival every day for at least one week. Sensory disturbances are closely related to ligation-induced changes in ipsilateral ION. The CCI-ION model provides an appropriate animal model for the study of TN mechanisms and treatments [1,2].

During model-making surgery, animals of both the TN model and sham model groups were randomly divided into two subgroups to get optogenetic or null virus injection immediately after model-making surgery. Sixteen rats were subjected to an optogenetic viral vector (AAV2-hSyn-hChR2(H134R)-EYFP; concentration: 1.9 × 10^13^ GC/µL; Korea Institute of Science and Technology, Seoul, South Korea) injection contralateral to the ION. In contrast, the remaining 16 were subjected to a null virus (AAV2-hSyn-EYFP; concentration: 5 × 10^12^ GC/µL; Korea Institute of Science and Technology, Seoul, South Korea) injection. Vectors were intracranially injected into the nucleus accumbens core (NAcc; AP, bregma, +1.2 mm; ML, midline, +1.4 mm; DV, skull, −6.8 mm) [65] with a stereotaxic apparatus under general anesthesia. Animals in the sham group were injected with either optogenetic virus or null virus into the NAcc. We injected an adeno-associated virus carrying the ChR2-EYFP fusion protein driven by the human synapsin (hSyn) promoter for the excitation of the neurons. The optogenetic virus and null virus were diluted with PBS at ratios of 1:8 and 1:5, respectively. Then, 2 µL of the virus was injected at a rate of 0.3 µL/min using a Hamilton syringe and an automatic micro-syringe pump. After injection, the needle was kept in the same place for 5 min to ensure virus absorption. It was then retracted very slowly. Then the animals were carefully returned to their cages and checked regularly for seven days.

### 4.4. Behavioral Testing

All behavioral tests were conducted one day before (baseline) model-making surgery, at every seventh day after the surgery, and with optic stimulation after optic fiber implantation. Behavioral tests were performed by an examiner who was unaware of the group of the animals.

#### 4.4.1. Air-Puff Test

The air-puff test was conducted by following the same procedures described in previous studies [2]. In brief, the animals were placed in a plexiglass cage and then, after getting habituated to the experimental room, a continuous puff of air was applied to the ipsilateral CCI-ION side of the face. The air puffs were delivered at an angle of 90°. We recorded and evaluated the psi level of the air puff at which the animals showed aggressive behaviors, like turning the head away or biting. Mechanical thresholds were evaluated and represented above 50% of the overall responses.

#### 4.4.2. Facial Cold Allodynia Score Test

The animals were placed in the same plexiglass cage described above. For this test, a few drops of acetone were placed on the vibrissal pad ipsilateral to the ION using a glass syringe, following which we counted the number of scratching/rubbing behaviors over the next 2 min. Body parts other than the face were excluded from this assessment [2]. 

#### 4.4.3. Von Frey Filament Test

The mechanical threshold level of the orofacial region was measure by von Frey filaments following the method described previously [2]. After habituation, the stimuli were administered with the filament by placing it near the center of the vibrissal pad of the ipsilateral CCI-ION. The lowest force (in grams) required to exhibit reactions such as immediate withdrawal, escape behaviors, attacking the filament, or asymmetric stroking of the face was recorded.

#### 4.4.4. Open Field Test

The open field test is an extensively used behavioral analysis method that can reflect the extent of characteristic emotional changes in response to the orofacial pain phenomenon. Excessive facial grooming behavior directly indicates the presence of orofacial pain whereas reduced activity time, decreased rearing events, and shortened distance traveled indicate stress due to pain sensation [66,67]. For the open field test, the animals were taken to the test room one by one and kept in the test field area, which was plain and illuminated and consisted of a 70 cm × 70 cm arena with 30 cm high walls. The area was cleaned and wiped with 95% ethanol before introducing the animal each time. The responses of the animals were recorded for 10 min for each time point on an overhead video recording camera which was placed vertically 1 m above the test field. The videos were analyzed later by using ToxTrac software [68]. Animals were placed at one corner of the open field arena and the following parameters were analyzed: number of explored areas, rearing times, active time, overall distance traveled, and pain-like behavior (facial grooming).

### 4.5. Optical Stimulation

We used a laser power supply with a wavelength of 473 nm (BL473T3-100, ADR-700D, Shanghai, China) and a waveform generator (Keysight 33511b-CFG001, Keysight, Santa Rosa, CA, USA) to regulate the waveform and pulse width of the laser. The laser’s intensity was set to 10 mW, the pulse width was set to 4 ms, and the pulses were set to 20 Hz [2,14,69]. A blue light was delivered via the optical fiber through a rotary joint patch cable that enables movement during testing.

### 4.6. Blocking of α-CGRP Receptor Pathway in the Trigeminal Ganglion

Before performing the extracellular recording with optic stimulation, animals from each group (TN-Opto, TN-Null, Sham-Opto, and Sham-Null) were again divided into two sub-groups of eight animals in each. In one group, the animals received an injection of α-CGRP (α-CGRP (8-37) (mouse, rat) trifluoroacetate salt, 1 mg/mL, BACHEM) as a calcitonin gene-related peptide (CGRP) receptor antagonist, while the animals in the other group received an injection of PBS. A total of 5 µL of α-CGRP or PBS was injected directly into the ipsilateral trigeminal ganglion (AP: −3.5 mm, ML: −3.6 mm, DV: −12 mm) before extracellular recording [70] and in vivo electrophysiology was performed after that. α-CGRP was injected at a rate of 0.3 µL/min using a Hamilton syringe and an automatic micro-syringe pump. After injection, the needle was kept in the same place for 5 min to ensure the absorption of CGRP, following which it was slowly retracted.

### 4.7. Extracellular Recording In Vivo

The extracellular recordings were obtained from the VPM thalamus (AP −3.5 mm, ML 2.8 mm, DV −6 mm [55]) using a single electrode under general anesthesia. Following a 20 min resting condition, we recorded thalamic neuronal spikes and firing rate during the pre-stimulation, stimulation (blue: 473 nm), and post-stimulation states in vivo. A glass-insulated carbon fiber microelectrode (catalog number: E1011-20, Carbostar-1, Kation Scientific, LLC, MN 55414, Minneapolis, MN, USA) was used for recording in the thalamus. Recordings were obtained for 5 min during each stage, with a 5 min gap between stages. We chose a well-isolated cluster and recorded neuronal signals using a Digital Lynx SX (Neuralynx, Bozeman, MT, USA) data acquisition system along with Cheetah software. We digitized and bandpass-filtered at 40 kHz and 1 Hz–5 kHz, respectively. The sorting was conducted offline using Spike Sorter 3D (Neuralynx Inc., Bozeman, MT, USA). The neuronal discharge was evaluated in the contralateral VPM of the lesioned and sham animals. The rate histograms (spikes/s) of the lesioned animals under different optical conditions were analyzed using NeuroExplorer (Neuralynx Inc., Bozeman, MT, USA).

### 4.8. Analysis of the Bursting and Firing Rates

Activity in the thalamic neurons was divided into burst rates (bursts/s) and overall firing rates (spikes/s) in each optical stimulation condition using NeuroExplorer software (Neuralynx Inc. Bozeman, MT, USA) [2,14]. The activity was assessed for 5 min in each state: pre-stimulation, stimulation, and post-stimulation. We defined burst rates as a group of at least three spikes with a maximum interval of 4 ms between spikes, with an interval of 100 ms between bursts. We selected similar inter-spike interval histograms for comparison among the groups.

### 4.9. Optic Fiber and Guide Cannula Implantation

After seven weeks of model-making surgery, 12 animals from each of the TN (TN-Opto, TN-Null) and sham (Sham-Opto, Sham-Null) groups were positioned in a stereotactic frame following anesthesia induction. An optic fiber was implanted into the skull contralateral to the ION (AP: +1.2 mm, ML: +1.4 mm) to transmit laser pulses to the NAcc. The optic fibers (200 µm core, 230 µm outer diameter, numerical aperture of 0.48, hard polymer cladding type, Doric Lenses; Québec City, QC, Canada) were cut to a length of 6.7 mm to optimize the targeting of the NAcc. Dental cement was used to fix the fiber firmly in place (Ortho-jet Pound Package, Lang Dental, Wheeling, IL, USA).

For microdialysis purposes, four animals from each group (TN-Opto, TN-Null, Sham-Opto, Sham-Null) in which optic fiber was not implanted were taken to implant guide cannula. The stainless-steel guide cannula (CXGF-7; Eicom, Fushimi-Ku, Kyoto, Japan) was stereotaxically implanted contralateral to the CCI-ION (AP +1.2 mm, ML +1.4 mm, DV −6.8 mm) and fixed using two bone screws and dental cement. Until the in vivo microdialysis, the cannula was sealed with a dummy cannula (CXDF-7) to prevent cerebral fluid loss.

### 4.10. In Vivo Microdialysis

To observe the effect of optogenetic activation of NAcc on different neurotransmitters’ concentration levels in optic stimulation “ON” and “OFF” conditions, in vivo microdialysis was performed with an optogenetics-compatible microdialysis probe (CX-F-07–01; Eicom, Fushimi-Ku, Kyoto, Japan) in four rats of each group (TN-Opto, TN-Null, Sham-Opto, and Sham-Null). Before starting the microdialysis procedure, the dummy cannula was replaced with a dialysis probe with a semipermeable membrane in its tip. The probe consisted of inlet and outlet channels connected to a two-channel fluid swivel system (SSU-20, Eicom, Fushimi-Ku, Kyoto, Japan). Then, artificial cerebrospinal fluid (ACSF, Tocris Bioscience, Bristol, UK) was infused through the probe using an infusion syringe pump (ESP-32, Eicom, Fushimi-Ku, Kyoto, Japan) at a rate of 1 μL/min. In an anesthetized and unrestrained condition, microdialysis was performed in a testing cage. After an equilibrium period of 1 h, 10 μL of dialysate was collected every 10 min into vials containing 10 μL of acetic acid solution (40 mM) placed on an automatic fraction collector. The temperature of the fraction collector was regulated by an electronic cooler (EFR-82, Eicom, Fushimi-Ku, Kyoto, Japan). The samples were collected under three different conditions—pre-stimulation (0–10, 10–20 min), stimulation “ON” (20–30, 30–40 min), and post-stimulation (40–50, 50–60 min). The blue laser was off in pre- and post-stimulation conditions and on in the stimulation “ON” condition. Immediately after the samples were collected, they were stored at −80 °C until assay.

### 4.11. Detection of Extracellular Concentration Levels of GABA, Glutamate, Dopamine, Acetylcholine, and Citrulline

To detect the differences in the extracellular concentration levels of GABA, glutamate, dopamine, acetylcholine, and citrulline in the NAcc in the optic stimulation “ON” and “OFF” states, liquid chromatography/tandem mass spectrometry (LC/MS/MS) was used (Supplementary Method). L-Glutamic acid (PHR1107-1G, Sigma-Aldrich Co., St. Louis, MO, USA), γ-aminobutyric acid (03835-250MG, Sigma-Aldrich Co., St. Louis, MO, USA), dopamine hydrochloride (PHR 1090-1G, Sigma-Aldrich Co., St. Louis, MO, USA) acetylcholine chloride (PHR1546-500MG, Sigma-Aldrich Co., St. Louis, MO, USA), and L-citrulline (1133842-200MG, Sigma-Aldrich Co., St. Louis, MO, USA) were used as internal standards.

### 4.12. Histological Examinations

Immediately after performing behavioral tests with optogenetic stimulation, the rats were deeply anesthetized and transcardially perfused with PBS followed by 4% paraformaldehyde. The brains were extracted and fixed overnight in the same post-fixed solution, followed by dehydration in a 30% sucrose solution.

We embedded the brains in optimal cutting temperature (O.C.T.) compound (Tissue Tek^®^, Sakura, Torrance, CA, USA). Then, they were cryopreserved with liquid nitrogen and isopentane at −79 °C. A cryostat (Thermo Scientific, Waltham, MA, USA) was used to cut coronal sections of the brain (20 µm). The brain sections were incubated with 4′,6-diamino-2-phenylindole (DAPI) and mounted with coverslips for examination under a fluorescence microscope to observe the optogenetic and null virus expression. H&E staining was also done to confirm the anatomical location of the NAcc where the virus was injected and the placement of the optic fiber and guide cannula.

For double immunofluorescence labeling of FOS-positive cells and GABAergic neurons, the sections were washed with wash buffer (10 × tris buffered saline) and then blocked with blocking solution (10% goat serum albumin) for 1 h. Thereafter the sections were incubated overnight at 4 °C with primary antibodies (c-FOS, Abcam ab208942; GABA, Sigma-Aldrich, A2025). The next day the sections were washed with wash buffer and incubated with secondary antibody (Alexa Fluor 488) at room temperature for 2 h. The slides were washed again in PBS, mounted with Fluoroshield, coverslipped, and finally examined under a fluorescence microscope.

### 4.13. Statistical Analysis

Data were analyzed using GraphPad Prism (GraphPad Software version 8.4.2, Inc., San Diego, CA, USA) and represented as the “mean value ± standard deviation”. We performed either an unpaired *t*-test, two-way analysis of variance (ANOVA) with Tukey’s *post hoc* test, or a repeated-measures ANOVA, depending on the conditions of the experiment. The behavioral tests were assessed based on the mean values for each of the three optical states. Unpaired *t*-tests were used to compare the firing rates between the TN and sham-operated animals and also between the stimulation “OFF” and “ON” conditions in the open field test. A *p*-value of <0.005 was considered statistically significant in every case.

## 5. Conclusions

In fine, this study showed that stimulation of the NAcc manipulated the functional connections between areas involved in pain circuitry by altering the release of neurotransmitters and dopaminergic pathway, thus deflecting the behavioral outcomes and VPM thalamic discharge in the trigeminal neuropathic pain condition. Therefore, our results provide evidence relating to the potential involvement of the NAcc in trigeminal pain modulation that could be efficacious in understanding and exploring the role of the NAcc in future studies and in using it in the field with regard to TN pain management.

## Data Availability

The data that support the findings of this study are available on request from the corresponding author. The data are not publicly available due to their containing information which could compromise the privacy of research participants.

## Supplementary Materials

The following are available online at https://www.mdpi.com/article/10.3390/ijms22168421/s1.
