# Supplementary material for: Stimulating GABAergic Neurons in the Nucleus Accumbens Core Alters the Trigeminal Neuropathic Pain Responses in a Rat Model of Infraorbital Nerve Injury
Source: Int J Mol Sci. 2021 Aug 5;22(16):8421. doi: 10.3390/ijms22168421 (PMC8395143; doi:10.3390/ijms22168421)
Supplement: Supplementary file 1 [file ijms-22-08421-s001.zip › ijms-1309386-supplementary-v2.pdf]

Supplementary Material

Supplementary Figure S1

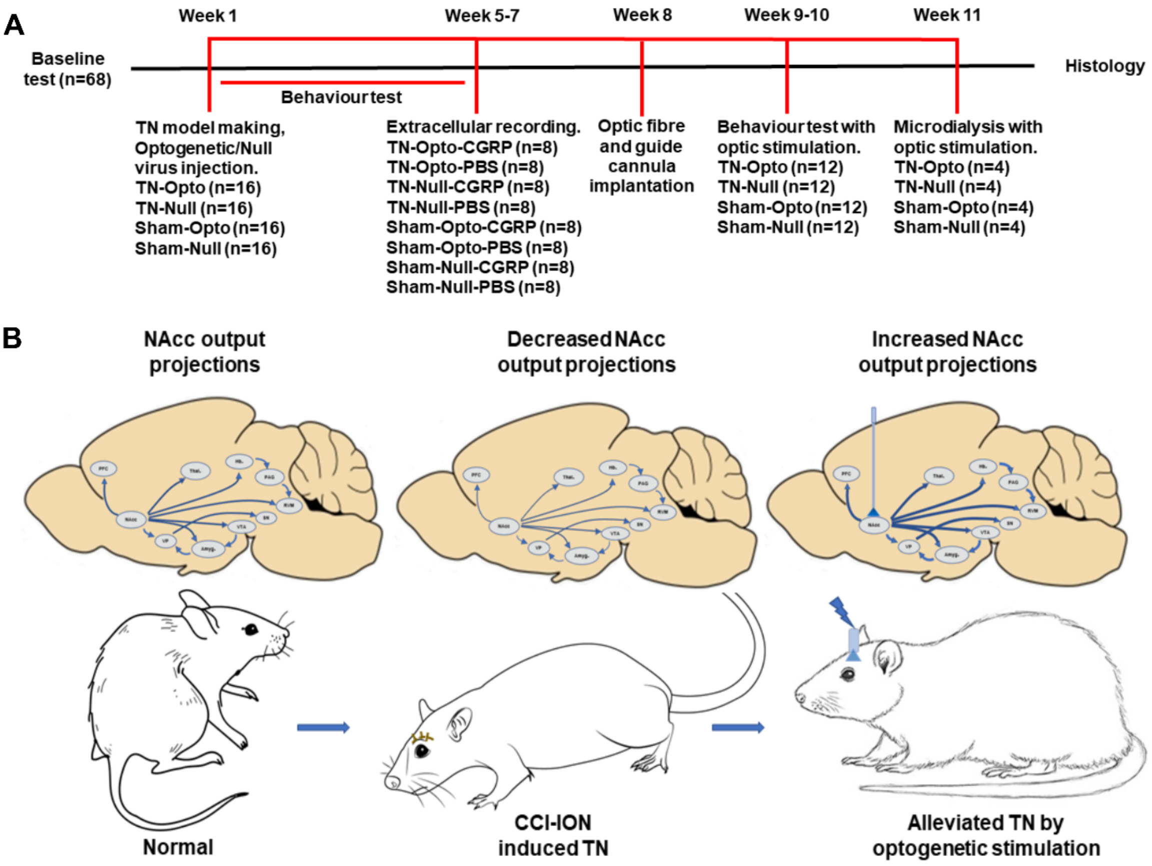

Experimental protocol and timeline. (a) Experimental timeline. (b) Output projections from NAcc to different brain regions involved in pain processing pathway. PFC = Prefrontal cortex, VP = Ventral pallidum, Thal. = Thalamus, Hb. = Habenula, PAG = Periaqueductal gray, RVM = Rostral ventromedial, SN = Substantia nigra, VTA = Ventral tegmental area, Amyg. = Amygdala.

Supplementary Figure S2

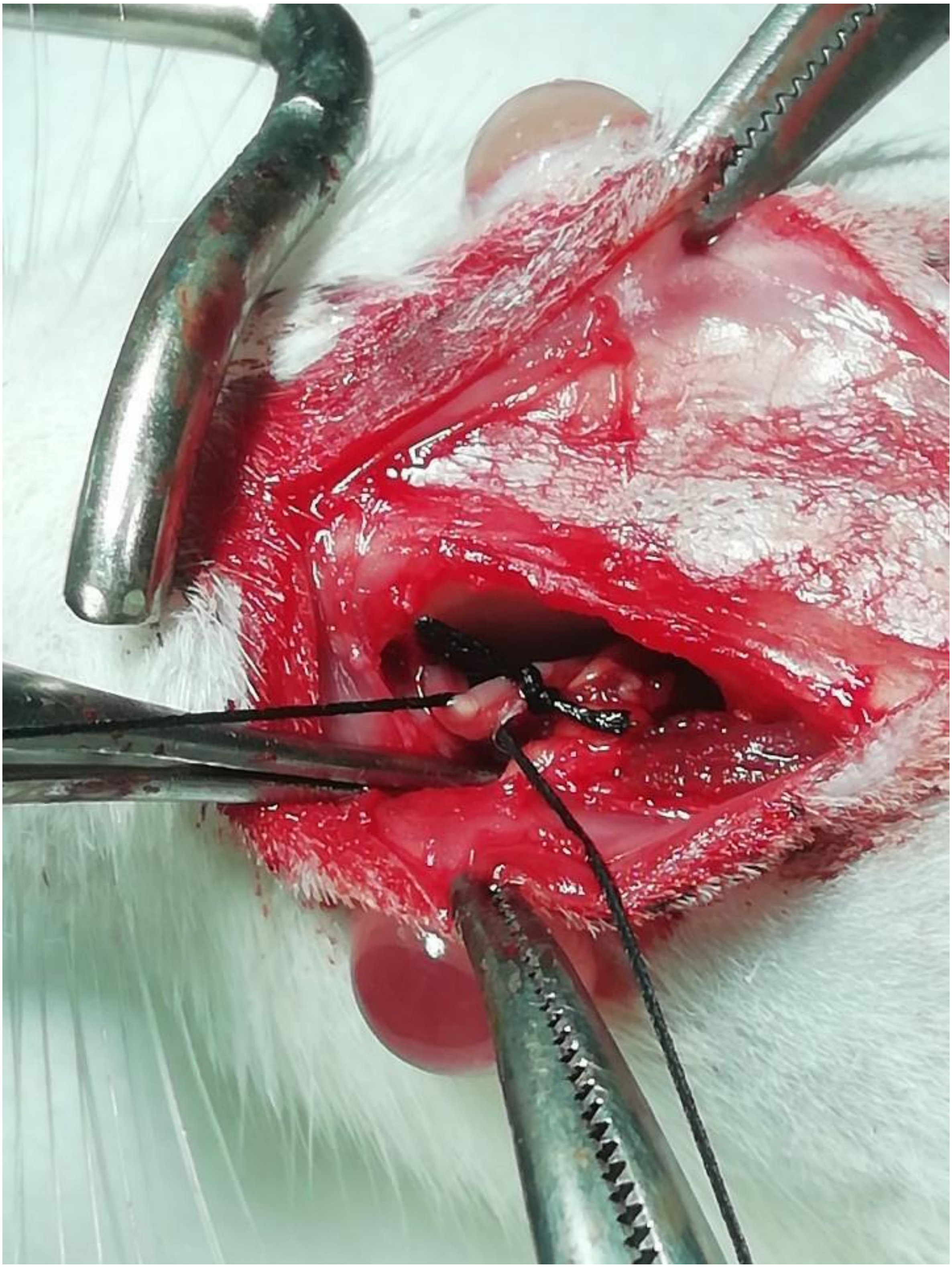

Generation of chronic constriction injury of infraorbital nerve. General anesthesia in animals were accomplished with an intraperitoneal (i.p.) injection of a mixture of 15 mg/kg Zoletil (Zoletil50®, Virbac Laboratories, Carros, France) and 9 mg/kg Rompun (Rompun®, Bayer, Seoul, South Korea) in saline. After that they were mounted onto the surgical field in a prone position. The skin above the eye was then shaved, and then the animals were placed in a stereotaxic frame. To prevent any damages associated with drying of the eyes, ophthalmic ointment was applied to the cornea. A skin incision of approximately 7 mm in length was made along the curve of the frontal bone in the anterior-

posterior direction, 2 mm above the left eye. Moving laterally, the fascia and muscle were gently separated from the bone using a periosteal elevator. The infraorbital nerve (ION) could be observed on the maxillary bone following retraction of the eye. Once revealing the ION, we prepared for ligature placement by gently freeing approximately 8 mm of.

the ION from the surrounding connective tissue. The ION was stretched slightly using a blunt needle with a curved head for ligature placement. The two ligatures were gently placed 3-4 mm apart, following which they were tightened until the ION was barely constricted. Finally, the incision above the eye was sutured with silk (3-0).

Supplementary method

LC/MS/MS analysis procedure: Chromatographic analysis was carried out on an Agilent 1100 high-performance liquid chromatography (HPLC) system (Agilent Technologies, Santa Clara, CA, USA) comprising a G1322A degasser, a G1311A quaternary pump, a G1313A well-plate autosampler, and a G1316A thermostated column compartment. To detect mass spectrometry, a G1946D mass spectrometer (Agilent Technologies, CA, USA) consists of an electrospray source interface was used. To identify LC/MS/MS, Agilent ChemStation (version B.02.01) was utilized for data acquisition and analysis. A column of Agilent XDB-C18 (3.0 mm x 50 mm; id, 1.8 μm) was used to achieve chromatographic separation and eluted with a mobile phase of acetonitrile: 0.1% aqueous solution of formic acid (24:76, v/v) at a flow rate of 0.3 mL/min. The temperature of the column was maintained at 25°C, the autosampler was maintained at 4°C and the volume was 5 μL. Analysis time for each sample was 4.5 minutes. The HPLC system, via an electrospray ionization (ESI), was connected to the mass spectrometer. The ESI mass conditions were optimized in the mode of negative ion detection. Selected ion monitoring was used, and the fragmentation transitions were m/z 511.1 for curculigoside and m/z 579.1 for naringin.
